# Retrospective study of tetanus in 18 dogs—Causes, management, complications, and immunological status

**DOI:** 10.3389/fvets.2023.1249833

**Published:** 2023-11-02

**Authors:** Stefanie Dörfelt, Christine Mayer, Georg Wolf, Reinhard K. Straubinger, Andrea Fischer, Katrin Hartmann, Rene Dörfelt

**Affiliations:** ^1^Ludwig-Maximilians-University (LMU) Small Animal Clinic, Centre for Clinical Veterinary Medicine, Ludwig-Maximilians-University (LMU), Munich, Germany; ^2^Department of Veterinary Sciences, Institute for Infectious Diseases and Zoonoses, Ludwig-Maximilians-University (LMU), Munich, Germany

**Keywords:** *Clostridium tetani*, canine, outcome, tetanospasmin, tetanus toxoid, treatment, vaccination

## Abstract

**Objective:**

Tetanus is a severe neurologic disease caused by *Clostridium tetani*, resulting in spastic paralysis. Canine tetanus is associated with serious complications such as aspiration and a high mortality rate of up to 50%.

**Materials and methods:**

Medical records of all dogs diagnosed with tetanus over 8 years (2014–2022) were analyzed for severity grade, treatment protocols, nutritional management, and complications, as well as outcome, vaccination, and antibody production in some dogs. No medical records were excluded. Normality was analyzed by the D'Agostino–Pearson test. Parametric, normally distributed data were presented as mean ± standard deviation. Non-parametric, non-normally distributed data were presented as median (m) and range (minimum–maximum). The association between tetanus grade, progression of diseases, and duration of hospitalization was analyzed using the *t*-test, Mann–Whitney U test, and Kruskal–Wallis test. A *P* ≤ 0.05 was considered significant.

**Results:**

Eighteen dogs were identified. Most affected dogs were classified into severity grade II (66.7%, 12 of 18). Clinical signs deteriorated in 55.6% of dogs (10 of 18). A source was identified in 88.9% of dogs (16 of 18). Nine dogs required surgical wound revision. A percutaneous endoscopic gastropexy tube was placed in 83.3% of dogs (15 of 18) for nutritional support. Medical treatment included metronidazole, methocarbamol, and combinations of different sedatives adapted to the patient's requirements. Tetanus antitoxin was used in 72.2% of dogs (13 of 18) without reported adverse events. The survival rate was 88.9% (16 of 18). Complications, such as hypertension, aspiration pneumonia, and laryngeal spasm occurred in 12 of 18 dogs. Median hospitalization time (8 days; range 0–16 days) was associated with the maximum tetanus severity grade (p = 0.022). Rapid eye movement behavior disorder was observed in 72.2% of dogs (13 of 18). In 5 dogs, antibodies were measured after recovery, and in 4 of 5 dogs, no antibodies were detectable despite generalized tetanus disease. Vaccination with tetanus toxoid was performed in five dogs following the disease.

**Conclusion:**

In the present study, the mortality rate was lower than previously reported. Tetanus is still a life-threatening disease, but the prognosis may be good if adequate management and monitoring can be ensured.

## 1. Introduction

Tetanus commonly presents as a life-threatening neurologic disease. This clinical syndrome is caused by tetanospasmin, a neurotoxin (TeNT) released by *Clostridium (C.) tetani*, a ubiquitous, anaerobic, spore-forming, gram-positive bacterium, which replicates mainly in infected wounds (1, 2). In a suitable anaerobic environment, such as tissue necrosis and cell lysis, *C. tetani* produces the toxin TeNT, which attaches to the presynaptic membrane of demyelinated nerve endings and migrates retrogradely to the neuronal cell body of inhibitory interneurons within the central nervous system (3). The toxin cleaves synaptobrevin, a vesicle-associated membrane protein required for the release of inhibitory neurotransmitters, such as glycine and gamma-aminobutyric acid (GABA) (4). The consequence is the lack of release of these inhibitory neurotransmitters, resulting in overstimulation of motor neurons with focal or generalized increased muscle tone that can worsen with excitement and other stimuli (5). The sensitivity to TeNT differs between species, with dogs and cats being much less sensitive compared with horses and humans. The minimal lethal dose of tetanus toxin *via* the intramuscular route of inoculation is 0.2 ng/kg in horses and humans. As the minimal lethal dose is higher in dogs (150 ng/kg) and cats (600 ng/kg) (6), vaccination is currently recommended in humans and horses but not in dogs and cats (7).

The most common initial clinical signs in affected dogs include oculofacial signs with trismus, risus sardonicus, dysphagia, and vomitus (5, 8). In more than half of the dogs, clinical signs progress to generalized rigidity with tonic, involuntary, prolonged muscle contractions with or without autonomic dysfunction (3, 5). Potential complications during the disease are (I) respiratory compromise due to laryngeal spasm, aspiration pneumonia, and central respiratory arrest; (II) autonomic dysfunction with brady-/tachycardia, hypotension, and urine retention; and (III) others, such as malnutrition, hyperthermia, hiatal hernia, megaesophagus, and urinary tract infection (5, 9, 10). Coxofemoral luxation and systemic inflammatory response syndrome may occur on rare occasions (5, 9, 11). Reported mortality rates in dogs are between 8% and 50% (5, 12–15).

Treatment strategies include the removal of the source of infection (if still ongoing), antitoxin, adequate antimicrobial therapy, control of rigidity and tetanic spasms with muscle relaxants and sedatives, and long-term intensive supportive care (5, 16). Any stimuli that could exacerbate spasms, e.g., pain, noise, manipulation, and light, should be avoided. Adequate analgesia is mandatory to control pain from muscle spasms and if procedures such as toe amputation are performed. Dogs that are dysphagic due to pharyngeal spasms and trismus, or dogs with deep sedation, need adequate nutritional support.

To date, no definitive antemortem laboratory test has been established to diagnose tetanus (15, 17). Before the onset and compatible clinical signs, history or suspicion of previous wound infection in the days or weeks prior usually indicate a clinical diagnosis of tetanus (1). Hematology and serum biochemistry panels are unspecific, often without any deviations, and only mild changes in the complete blood count, elevated creatine kinase, and increased serum creatinine levels can be observed (14, 18). Isolation of *C. tetani* from wounds often fails, as it requires anaerobic conditions and special culture media and may no longer be present on rare occasions (11). Measurement of serum antibodies to tetanus toxoid has been suggested to provide further support for the diagnosis of tetanus in case of positive results (1, 8). However, no information is available on the course of the production of antibodies in these dogs. In human medicine, a small quantity of TeNT already causes clinical disease but is insufficient to stimulate antibody production (7). Nowadays, the diagnosis of tetanus in dogs is still based on clinical signs and history.

This retrospective analysis aimed to evaluate the outcome of dogs with tetanus and summarize intensive care treatment protocols. The secondary aim was to describe the course of tetanus toxoid antibody production after the development of the disease and after vaccination in some of the dogs.

## 2. Materials and methods

Medical records of dogs diagnosed with tetanus that were presented between August 2014 and February 2022 to the Clinic of Small Animal Medicine, Center for Clinical Veterinary Medicine of the LMU Munich, were reviewed. Dogs with supportive history and characteristic clinical oculofacial signs consistent with tetanus, such as risus sardonicus, trismus, prolapse of the third eyelid with or without dysphagia, and generalized stiff gait, without evidence of other central nervous system or neuromuscular diseases, were included. Medical records were evaluated for signalment, age, body weight, presence of underlying source of infection, onset of clinical signs, clinical signs at presentation, bacteria isolated from wounds, surgical interventions, treatment protocols including sedation and muscle relaxation protocols, time points of placement of percutaneous endoscopic gastrostomy tube (PEG tube) during disease, course of disease, time until adequate food uptake and time when PEG tube was removed, duration of hospitalization, and complications. For the evaluation of clinical signs, an established tetanus severity classification system was used (Table 1) (5). Follow-up data were obtained on the presence of rapid eye movement sleep behavior disorder (RBD) (13). In addition, after disease and vaccination with subsequent antibody titer development, the course of tetanus toxoid antibody titer was evaluated. Antibody titers were controlled within 3 months after infection and 1 and 2 years after vaccination.

**Table 1:** Tetanus severity classification modified from Burkitt JM, Sturges BK, Jandrey KE, Kass PH. Risk factors associated with outcome in dogs with tetanus: 38 cases (1987–2005).

**Grade**	**Clinical signs**
I	Any or all of the following signs (ambulatory dogs): •Oculofacial signs: miosis, enophthalmus, *risus sardonicus*, erected ears, trismus •Hypersensitivity to noise, light, touch
II	Any or all of the following signs (ambulatory dogs): •Dysphagia •Stiff gait, sawhorse stance, erected tail •May have any or all grade I signs
III	Any or all of the following signs (recumbent dogs): •Muscle fasciculations or spasms •Seizures •Must have grade I or II signs
IV	Any or all of the following signs: •Autonomic signs (brady-/tachycardia, brady-/tachyarrhythmia; hypo-/hypertension, apnea, respiratory arrest) •Must have grade I, II or III signs

### 2.1. Data analysis

Statistical analysis was performed using commercial software.^1^ Normality was analyzed by the D'Agostino–Pearson test. Parametric, normally distributed data were presented as mean ± standard deviation. Non-parametric, non-normally distributed data were presented as median (m) and range (minimum–maximum). The association between tetanus grade, progression of diseases, and duration of hospitalization was analyzed using the *t*-test, Mann–Whitney *U* test, and Kruskal–Wallis-test. A *P* ≤ 0.05 was considered significant.

## 3. Results

### 3.1. Signalment of dogs with tetanus

Eighteen dogs aged 34.5 ± 40.6 months, with a median body weight of 25.0 kg (5.0–50.0 kg), met the inclusion criteria of the study (Supplementary File 1). Breeds included mixed-breed dogs (*n* = 6), Labrador Retriever (*n* = 2), and one each of American Staffordshire Terrier, American Pocket Bully, Border Collie, Collie, Boxer, Dutch Shepherd, Elo, Great Dane, Jack Russel Terrier, and Rhodesian Ridgeback. Six dogs were male intact, three dogs were male neutered, four dogs were female intact, and five dogs were female neutered.

### 3.2. Source of infection and isolation of *C. tetani*

In 16 of 18 (88.9%) dogs, a potential site of infection could be identified (Supplementary File 2). Nine dogs had a wound in one digit of the thoracic limb, five dogs had a wound in one digit of the pelvic limb, one dog had a wound at the proximal pelvic limb, and one dog developed clinical signs of tetanus during teething. In total, 8 of 18 (44.4%) dogs with nail injuries, 2 of 18 (11.1%) dogs with footpad wounds, and 4 of 18 (22.2%) dogs with digit lacerations could be identified at the time of presentation. Radiographic evaluation of the injured paws was performed in 12 cases. Radiographs showed osteomyelitis of one distal phalanx in 6 of 12 (50.0%) dogs, soft tissue swelling in 9 of 12 (75.0%) dogs, and a fracture of one distal phalanx in 3 of 12 (25.0%) dogs. In the remaining two dogs, the source of infections remained unknown.

In 8 of 18 (44.4%) dogs, samples from the infected site were submitted for bacterial culture (Supplementary File 2). In 2 of 8 (25.0%) dogs, no bacteria could be isolated during surgery (quality control swab). All dogs received antibiotic treatment at that time point. In the remaining six dogs, different bacteria including *Staphylococcus simulans, Staphylococcus pseudintermedius, Enterococcus faecium*, and *Citrobacter freundii* could be isolated (Supplementary File 2). *Clostridium tertium* was identified in one dog, and *Clostridium sporogenes* was identified in two dogs.

### 3.3. Clinical signs and progression

Overall, 2 of 18 (11.1%) dogs presented with tetanus grade I, and both dogs showed miosis, strabismus, risus sardonicus, one with mild trismus and one without trismus, and no dysphagia or stiff gait (Supplementary File 1). The majority of dogs (12 of 18, 66.7%) presented with tetanus grade II. Clinical signs in all these dogs included a combination of miosis, strabismus, enophthalmos, risus sardonicus, erected ears, and hypersensitivity to noise. All grade II dogs showed dysphagia and generalized stiff, but ambulatory gait. In total, 4 of 18 dogs (22.2 %) displayed signs consistent with grade III on presentation. In addition to signs of grade II, these dogs displayed generalized muscle fasciculations (*n* = 3) or muscle spasms (*n* =1).

A possible source of infection was reported in 13 of 18 (72.2%) dogs (in 4 dogs, no wound was identified; in 1 dog, no information was available). The median time from the occurrence of the wound to the first clinical signs was 6 days (range 1–27 days), and the median time to presentation was 12 days (range 4–28 days; Supplementary File 1). Clinical signs progressed after the presentation in 10 of 18 (55.6%) hospitalized dogs within a median time of 2 days (range 1–5 days; Supplementary File 1), with 6 dogs progressing to tetanus grade IV. In these six dogs, autonomic signs occurred, including ventricular arrhythmia (*n* = 2), bradycardia (<35 bpm; *n* = 3), tachycardia (> 150 bpm; *n* = 2), hypertension (systolic blood pressure range 189–220 mmHg; *n* = 4), and death of unknown cause in two dogs (Supplementary Files 1, 3).

### 3.4. Surgical treatment

Surgical wound revision was performed within 48 h after hospitalization of 9 of 18 (50.0%) dogs (Supplementary File 2). All nine dogs required amputation of the injured digit. All these digits showed soft tissue swelling, with purulent secretion (2 of 9, 22.2%) and panaritium (4 of 9, 44.4%). Radiologic evidence of osteomyelitis was observed in 6 of 9 (66.7%) dogs. The degree of amputation was determined by the visual or radiographic extent. In nine cases, the entire digit was amputated at the level of the metacarpophalangeal joint (1/9 dogs, 11.1%) or the distal level within the distal metacarpal bones via an osteotomy (8/9 dogs, 88.9%), all including removal of the sesamoid bones. Additionally, in one dog, the distal phalanx of two digits was amputated. Before starting surgery, in 8 of 9 dogs (88.9%), 1,000 I.U. tetanus antitoxin was injected locally along the line to healthy tissue. A soft-padded bandage was applied, the wound was cleaned, and the bandage was changed once daily.

### 3.5. Medical treatment and side effects

All but one dog (17 of 18, 94.4%) were hospitalized to manage the existing signs and to monitor clinical progression. One dog with initial tetanus grade I was treated as an outpatient. Standard management included shielding in a dark and quiet room with additional ear plugs or cotton wool placed into the outer ear canal to reduce auditory stimuli, cleaning the mouth and external ear canals once daily, application of eye drops, changing the dog's position every 4 h if the dog was recumbent, and manual bladder expression or placement of an indwelling urinary catheter if required. The individual sedation was increased and adapted to individual needs before handling and manipulation, depending on respiratory and cardiovascular status and side effects and reactions to the drug.

Standard medical treatment for all dogs included 10 mg/kg IV metronidazole (Fresenius Kabi AG, Bad Homburg Germany) every 8 h for at least 14 days and methocarbamol (Ortoton Recordali, Recordali Pharma GmbH, Ulm, Germany; dose, interval, and route adapted to the dog's requirement; Supplementary File 3).

Equine serum tetanus antitoxin (TAT, Tetanus Serum, WDT, Garbsen, Germany; Supplementary File 3) was used in 13 of 18 (72.2%) dogs. A test dose of 0.2 ml was injected subcutaneously 30 min before intravenous TAT application. No signs of hypersensitivity to TAT were observed in any dog. In 11 of 13 (84.6%) dogs, TAT was given once undiluted for 20 min as a slow intravenous injection (median 300 IU/kg, range 200–375 IU/kg). In two dogs, TAT was applied subcutaneously (1/13, 690 IU/kg) or intramuscularly (1 of 13, 250 IU/kg) by a veterinarian. No signs of hypersensitivity occurred in any dog.

Crystalloid fluids (Ringer-Lactat-Lösung nach Hartmann, B.Braun Vet Care; Sterofundin Iso, B.Braun Vet Care) were given intravenously as continuous rate infusion (CRI) to prevent dehydration and ongoing losses and fulfill maintenance requirements in all hospitalized dogs. A central venous catheter (CVC) was placed in 61.1% of the cases (11 of 18). Acepromazine was used for baseline sedation in all but four dogs. One of these four dogs had tetanus grade I (case No. 11), and no sedation was necessary. For the other three dogs, parenteral acepromazine product was not available on the German market at that time. Acepromazine was given as CRI combined with intermittent boli (*n* = 9) or as repeated boli every 6 to 8 h (*n* = 5; Supplementary File 3). Other drugs included in the sedation protocols were dexmedetomidine CRI (*n* = 9), midazolam CRI (*n* = 2), and opioids (fentanyl, butorphanol, and methadone) administered as a CRI (*n* = 4) or as an intravenous bolus (*n* = 14) and propofol CRI (*n* = 4) or bolus (*n* = 4). In the cases of a propofol CRI, intermittent boli were injected for the adaptation of sedation. In 44.4% of cases (8 of 18), different CRIs had to be combined to achieve adequate sedation and muscle relaxation (Supplementary File 3). Additional treatment included antibiotics (*n* = 11; 61%), such as amoxicillin/clavulanic acid alone (*n* = 6; 33%), amoxicillin/clavulanic acid in combination with marbofloxacin (*n* = 3; 17%), and ampicillin alone (*n* = 2; 11%) if concurrent infection or pneumonia was present; antiseizure drugs, such as phenobarbital (*n* = 3), gabapentin (*n* = 3), or levetiracetam (*n* = 5) for sedation; and antiemetic and gastro-protectant drugs, such as metoclopramide (*n* = 8), maropitant (*n* = 12), ranitidine (*n* = 2), omeprazole (*n* = 7), and chlorphenamine (*n* = 2), and magnesium sulfate (*n* = 2).

If additional sedatives besides acepromazine were started, a small test dose (half of the lowest recommended dose) was given intravenously, and side effects were evaluated. In one dog (case No. 7), escape beats and bradycardia (<35 bpm) were provoked by administration of 5 μg/kg buprenorphine and did not resolve after discontinuation. In another dog (case No. 10), respiratory depression was provoked by 1.5 μg/kg dexmedetomidine IV, which necessitated intubation and mechanical ventilation for 30 min. Dysphoria was observed after the application of benzodiazepines in two dogs. In two other dogs, hypoxemia occurred during the administration of propofol CRI, which was corrected by oxygen supplementation (flow by, nasal oxygen tube). In one of these dogs, hypoxemia was no longer evident after discontinuation of propofol. In the second dog, hypoxemia occurred due to brachycephalic syndrome (case No. 12) and deteriorated with propofol. After injecting dexmedetomidine CRI at a minimal dose, one dog (case No. 12) vomited. A generalized body tremor occurred with intravenous butorphanol administration (0.2 mg/kg) in another dog (case No. 14). The tremor stopped with midazolam treatment. One dog (case No. 7) started vomiting during metoclopramide CRI, which was given due to its prokinetic properties. The vomiting stopped with the subcutaneous administration of metoclopramide.

### 3.6. PEG tube placement and nutrition

In 83.3% of dogs (15 of 18), a PEG tube with additional gastropexy (PEG Feeding Tube PT20XL, Smith Medical 13/15; Freka PEG CH/Fr 15 ENFit, Fresenius Kabi AG 2/15) was placed under general anesthesia for enteral nutrition within 48 h after presentation due to dysphagia, decreasing the risk of aspiration during feeding (Supplementary File 3). In 7 of 8 dogs that had wound surgery, this procedure was combined with surgical wound management.

The PEG tube was cleaned daily with hypochloric acid (Vetericyn, Ecuphar) or polyhexanide (Prontovet Gel, B. Braun Vet Care) and covered with gauze. Nutrition *via* PEG tube was started 24 h after placement to fulfill one-third of the resting energy requirement (RER). The tube insertion site was controlled macroscopically in all cases and also *via* ultrasonography in 11 of 15 dogs (cases 7 to 18, 73.3%). Gastropexy sutures were left in place for a minimum of 10 days. As soon as an improvement in clinical signs was noticed and the dog was ambulatory again (in tetanus grade III and higher), oral water and food uptake were encouraged gradually. For this, a few drops of water were given into the mouth to evaluate the presence of adequate swallowing. When no salivation or vomiting occurred and the dog swallowed the water without any problem, the amount of water was increased slowly. If no salivation or vomiting was observed, a small amount of food was given and the amount was stepwise increased. The mean duration of spontaneous and physiologic oral food intake (physiologic mouth opening, normal swallowing, no salivation, no regurgitation, or vomiting) was 17.6 ± 6.7 days (recorded in 11 of 18 dogs), and the time of the removal of the PEG tube was 23.8 ± 6.4 days (recorded in 13 of 18 dogs). The earliest time for the removal of the PEG tube was 14 days after placement.

The removal of the PEG tube was performed in non-sedated dogs in a standing position in all but three dogs (12 of 15). In two dogs (case Nos. 15 and 18), the low-profile PEG tube was removed endoscopically under general anesthesia. For one dog (case No. 17), sedation was needed for removal. After removal of the PEG tube, water was withheld for 12 h and food was withheld for 24 h.

### 3.7. Complications

Non-invasive, oscillometric blood pressure recordings were available in 12 of 18 (66.7%) dogs (Supplementary File 3). In 4 of 12 (33.3%) dogs, severe hypertension with a mean systolic blood pressure of 201 mmHg (189–220 mmHg) was developed and treated with amlodipine once or twice daily. The remaining eight dogs were normotensive, or only mild increases in blood pressure occurred (systolic blood pressure range 120–156 mmHg). None of the dogs was hypotensive.

Aspiration pneumonia was a complicating feature in 5 of 18 dogs and was treated with beta-lactam antibiotics (amoxicillin/clavulanic acid and ampicillin) or fluoroquinolone (marbofloxacin). One of these dogs (case No. 7) developed moderate hypalbuminemia (18.2 g/l), and a parenteral amino acid solution (Aminoplasmal B. Braun 10% E, B. Braun AG) was administered.

In 16.7% of the dogs (3 of 18), life-threatening laryngeal spasms occurred due to excitement during examination (*n* = 1), manipulation for premedication for anesthesia (*n* = 1), and after recovery from anesthesia (*n* = 1), necessitating orotracheal intubation. The last dog also developed swelling at the tongue and larynx and required oxygen supplementation *via* a nasal tube. In all three dogs, aspiration pneumonia was observed.

In two dogs (case Nos. 1 and 7), a low heart rate and escape beats, suspected to be triggered by opioid administration, occurred.

In total, 15 of the 18 dogs received nutritional support *via* PEG tube. Reduced gastrointestinal motility was evident in 3 of 15 dogs. After 4 h of feeding twice, the amount given could be removed from the stomach via a PEG tube. In these dogs, metoclopramide CRI or subcutaneous bolus injection was initiated.

Complications such as urine retention (*n* = 2), bacterial cystitis (*n* = 1), and disseminated intravascular coagulation due to initial hyperthermia (*n* = 1) were further observed during hospitalization.

### 3.8. Duration of hospitalization

The median hospitalization time of the 16 surviving dogs was 8 days (0–26 days; Supplementary File 1). The two non-survivors were hospitalized for 3 days. No difference in hospitalization time was observed for dogs with different initial tetanus grades (*p* = 0.163). However, a significant association between maximum tetanus grades and duration of hospitalization was observed in surviving dogs (*p* = 0.022). Dogs with a maximum tetanus grade III or IV had a significantly longer hospitalization time (18 days; 5–26 days) compared with dogs with grades I to II (4 days; 0–9 days; p = 0.004). Hospitalization time in the eight dogs with progression in their tetanus severity grade was also longer (18.5 ± 6.7 days) compared with those eight dogs without progression (4.5 ± 2.9 days; *p* < 0.001). Time to improvement in clinical signs was available for 13 of 18 dogs that were successfully treated. The median time to first improvement in clinical signs was 11 days (range 4–15 days).

### 3.9. Outcome

The 28-day survival rate was 88.9%. In total, 17 of the 18 dogs were discharged (94.4%). Respiratory complications occurred in 5 of 18 (27.8%) dogs (aspiration pneumonia, upper airway obstruction) but were managed successfully and had no negative impact on the outcome as described elsewhere (9). Two dogs with grade III tetanus died, one in the hospital (death before discharge, case No. 3) and the other one at home, after being discharged at an early stage of the disease due to the owner's request (1 of 18, case No. 5). This dog was only hospitalized for 3 days and showed progressive signs. The two non-survivors and three surviving dogs did not receive TAT. Necropsy was not performed in any of the non-surviving dogs.

### 3.10. Development of rapid eye movement sleep behavior disorder

In 13 of 18 (72.2%) dogs, the occurrence of rapid eye movement sleep behavior disorder (RBD) was already documented during hospitalization. In all 13 dogs, RBD was transient and began on the first day after presentation and resolved within weeks to months after recovery from generalized tetanus. RBD ranged from mild (*n* = 7) to severe (*n* = 6). In mild cases, probable RBD presented with facial twitching, jaw chomping, and rapid eye movements during sleep (movements under the eyelids or movement of the head). In severe cases, dogs showed episodes with running movements of the limbs, paddling, biting objects lying around them (e.g., towels), jaw chomping, and growling. Episodes could be interrupted. Immediately after approaching these dogs, they woke up and the episodes stopped. No salivation or urination occurred during these episodes. In one dog (case No. 7), these episodes lasted for 3 h and resembled generalized tonic–clonic epileptic seizures. The dog did not wake up when approached. It received levetiracetam (30 mg/kg IV) and phenobarbital (5 mg/kg IV) once, which intensified the episodes. During this period, electroencephalography was performed, and no epileptiform paroxysmal discharges could be detected, classifying these episodes as non-epileptiform RBD.

### 3.11. Tetanus toxoid antibodies and vaccination

In five dogs, antitoxoid antibody production was evaluated after a successful recovery from tetanus disease (case Nos. 7, 10, 12, 16, and 18). Three of these five dogs recovered from tetanus grade IV (case Nos. 7, 10, and 12), one dog from tetanus grade III (case No. 18), and one dog from tetanus grade II (case No. 16). Serum tetanus toxoid antibody concentrations were measured 36–149 days after initial presentation (lateral flow assay, Miprolab Microbiologic Diagnostic, Göttingen, Germany). The test evaluates antibody titer against tetanus neurotoxin and is not species-specific. The matching of international units was performed with equine tetanus reference antitoxin from the National Institute for Biological Standards and Control, UK. The results were reported as “+” (equivalent to 0.1–0.9 IU/ml; Miprolab Microbiologic Diagnostic, Göttingen, Germany; (19)) in one dog (case No. 7) and “–” (no antibody titer present, equivalent to <0.1 IU/ml) in four other dogs (case Nos. 10, 12 16, and 18). In line with current recommendations in human medicine recommending vaccination after recovery from disease,^2^ and in line with the owners' wish to protect their dogs from this deadly disease, all five dogs were offered vaccination. The dogs were then vaccinated subcutaneously with tetanus toxoid (Equilis^®^ Te, tetanus toxoid 40 FL, MSD, Germany) (Table 2). In horses, an intramuscular route is recommended,^3^ whereas, in ruminants and small ruminants, tetanus vaccination is performed via a subcutaneous route.^4^ A subcutaneous route was chosen for dogs in the present study. Before vaccination, informed consent was obtained from the pet owners and documented in the medical records. In line with the recommendations in horses, ruminants, and humans, a second vaccination was performed in each dog after 4 weeks. Tetanus toxoid antibodies were measured 4 weeks and 1 year after the first vaccination in all dogs (*n* = 5, case No. 7, 10, 12, 16, 18) and 2 years after the first vaccination in two dogs (*n* = 2, case No. 7, 10) (Table 2). In all dogs, serum antitoxoid antibody concentration increased. Antibodies were still present after 1 year in all five dogs (*n* = 3 with ++, equivalent to 1.0–5.0 IU/ml, case No. 7, 10, 16; *n* = 2 with +++, equivalent to > 5.0 IU/ml, case No. 12 and 18) and were measured after 2 years in two dogs (*n* = 1 with ++, equivalent to 1.0–5.0 IU/ml, case No. 7; *n* = 1 with +++, equivalent to > 5.0 IU/ml, case No. 10, Miprolab Microbiologic Diagnostic, Göttingen, Germany). Adverse effects of vaccination, such as local pain and swelling at the injection site, fever, and anorexia, were observed in one dog (case No. 10). These adverse effects were treated with an anti-histaminic drug (cetirizine 1 mg/kg q 12 h, PO) and metamizole (50 mg/kg q 8 h, PO) for 3 days. Two dogs had no side effects. In two other dogs, this treatment was initiated prophylactically before or immediately after vaccination. One of these dogs developed pain at the injection site and was anorectic for 1 day (case No. 12).

**Table 2:** Tetanus toxoid antibody production after tetanus and vaccination in four dogs.

**Case No**.	**Antibody titer after tetanus**	**Timepoint of measurement of antibody titer and first vaccination after presentation (days after presentation)**	**Antibody titer after vaccination**		
			**Four weeks after first vaccination (**+ **second vaccination was performed)**	**One year after first vaccination (no further vaccination)**	**Two years after first vaccination (no further vaccination)**
7	+	109	++	++	++
10	–	149	+	++	+++
12	–	36	+	+++	Not yet performed^∫∫^
16	–	47	++	++	Not yet performed ^∫∫^
18	–	53	+	+++	Not yet performed ^∫∫^

Tetanus toxoid antibodies^*^.

- → no antibodies detected.

+ → 0.1–0.9 IU/ml.

++ → 1.0–5.0 IU/ml.

+++ → >5.0 IU/l.

^*^Miprolab Microbiologic Diagnostic, Göttingen, Germany, 19; ^∫∫^2 years not finished.

## 4. Discussion

The present retrospective study evaluated the clinical course and outcome of 18 dogs with tetanus. The survival rate (88.9%) in the present study is in the upper range of previous studies (12–92%) (5, 12–15). In the current study, dogs were managed intensively with individually adapted treatment protocols, intense monitoring to recognize any deterioration as early as possible, and early introduction of enteral nutrition. Despite this intensive care, two dogs died. In previous case reports and studies, dogs with generalized tetanus and higher severity scores, especially with autonomic dysfunction and respiratory complications (classification ≥ III upon admission) were more likely to have a poor outcome compared with those with less severe signs (5, 10, 14). The two non-surviving dogs in the present study had a tetanus severity score of III without obvious autonomic dysfunctions. Respiratory complications, which were associated with poor outcomes in previous studies, were also not evident in these two cases (10, 12, 14).

The potential source of infection was identified in 16 of 18 dogs with 14 dogs having paw lesions. Some were obvious and others were challenging to detect. Radiographic evidence of osteomyelitis was a frequent finding, which highlights the need for radiographic evaluation of injured digits. Therefore, clinicians should screen dogs with tetanus thoroughly for any paw or digit lesions, including radiographs (Supplementary File 2). Different sources of *C. tetani* infection, such as deep penetrating wounds (e.g., interdigital abscesses, bite wounds), broken or deciduous teeth, omphalitis, or surgical site infection, are documented in the veterinary literature (12, 15, 20). As the infection could originate from a small puncture wound and can no longer be visible when signs of tetanus develop, a missing source of infection does not rule out tetanus if typical clinical signs are present (9).

The data in this study highlight that tetanus frequently presents initially as a progressive disease. Signs of tetanus occurred in the reported cohort within a median time interval of 6 days after a wound was observed. After injury, the previously reported range of onset is 3 to 18 days (1, 5, 12). Other studies reported that tetanus can progress to complete body rigidity within 2 to 3 days after the initial hospitalization (12). In our cohort, tetanus progressed in 55.6% of the dogs for a median time of 2 days. Progression to grade III was observed in 17%, and progression to grade IV was observed in 33% of the dogs, which agrees with previously reported progression to grade III or IV with rates of 45–50% (5, 13). In the present study, more dogs with surgical treatment progressed to a higher severity score (6 of 9 dogs) compared with dogs without surgery (3 of 9 dogs). Surgical stimulation is a potential risk factor that could not be determined and is not evaluated in the present study.

In all dogs, specific treatment protocols, which were adapted to the specific needs of the individual dog, were used in combination with standard baseline medication including acepromazine, metronidazole, and methocarbamol. Equine serum TAT was given intravenously to 11 of 18 dogs and 2 dogs by another route (intramuscular, subcutaneous). The TAT which was used in our dogs is a purified high-titer antiserum which is obtained from horses immunized with *C. tetani* (9). The application of TAT aims to neutralize unbound toxins that might spread hematogenously after wound infection, but its use is still discussed controversially, and its effect is unproven (9, 16). No statistically significant difference in survival, severity, and duration of signs is reported between dogs treated with TAT and those not receiving TAT. There is no reported relationship between the timing of TAT administration, clinical course, and outcome (5, 12, 15). In 5 of 18 dogs, no TAT was given (case Nos. 3, 4, 5, 7, and 8). Due to the retrospective nature of this study, the cause for this decision could not be determined for earlier cases (case Nos. 3 and 4). In one dog (case No. 5), a wound could not be detected, and the timetable between a potential infection and presentation at the clinic was unknown. In two other dogs (case Nos. 7 and 8), the time between the wound (case No. 7) and potential wound (case No. 8) was 28 days and 20 days, respectively. For these cases (case Nos. 5, 7, and 8), TAT was not given, as there was a high risk that the tetanus toxin was already internalized. TeNT attaches to the presynaptic membrane of nerve endings after wound infection or hematogenous spread and internalizes and migrates retrogradely within axons to the neuronal cell body, making TeNT not accessible to TAT once it is internalized (18). After proximal and transsynaptic migration, TeNT is localized and exerts its effect on the gray matter of the central nervous system (e.g., spinal cord, brainstem) in inhibitory interneurons, interfering with neurotransmitter release. Passive immunization with TAT has limited effects, as the large immunoglobulin TAT does not cross the blood–brain barrier due to its size (9). Intrathecal administration of TAT could bypass the blood–brain barrier. In dogs and horses, intrathecal administration is reported (21–23). However, conflicting evidence of intrathecal TAT compared with other routes exists in human medicine. No definite benefit over parenteral administration is proven in human patients (24–27). Even though we observed a lower mortality rate, it is of interest that two dogs that died had not received TAT (case No. 3 and 5). The effect of parenteral TAT administration could not be evaluated in the present study due to the low mortality rate. The fatal outcomes were probably not related to the lack of TAT application.

Antibiotic therapy with coverage against anaerobic bacteria is commonly recommended for the treatment of tetanus to address any potential ongoing infection with *C tetani*. Nevertheless, deep wound infections and osteomyelitis might not be amenable to antibiotic treatment alone and can also require a surgical approach. Penicillin G has been suggested for a long time to be the antibiotic of choice against anaerobic gram-positive bacteria (15). It is documented that metronidazole penetrates the tissue with vascular compromise more effectively (15). People treated with metronidazole require lower doses of muscle relaxants and sedatives compared with people treated with penicillin G (28). In addition, potential GABA antagonistic properties of penicillin G are discussed to potentiate the effects of tetanospasmin, which might worsen rigidity and reduce the effect of benzodiazepines (29, 30). However, metronidazole can also exert neurotoxic effects, and signs of tremor, rigidity, ataxia, head tilt, and nystagmus can potentially occur if given in high doses or for a prolonged time period (31), although this has so far not been described in dogs treated for tetanus. However, caution should be exerted when metronidazole is administered at recommended dose (31).

In addition to resting in quiet surroundings and minimization of pain and manipulations, adequate sedation and muscle relaxation are important therapeutic goals in patients with tetanus. Acepromazine was used for baseline sedation and muscle relaxation in 14 of 18 dogs, and none of the dogs developed adverse effects from acepromazine. The two dogs with lethal outcomes did not receive acepromazine. In contrast with the result of a previous study, these findings reported a decreased survival of dogs treated with acepromazine (14). This finding could not be confirmed in the present study as most dogs were treated with acepromazine. A potential cause for worse outcomes in the previous study could be an institution of acepromazine treatment, especially in more severely affected dogs. As mentioned above, for the management of the dogs in the present study, individually adapted sedation and analgesic protocols were used, depending on individual efficacy and observed adverse effects. Typically, dogs were managed by clinicians who were dedicated at all hours to those dogs with tetanus, with additional support from the emergency and critical care team to be able to titrate medication and recognize complications in time. Magnesium sulfate was only used in two dogs in the present case series. In human studies, magnesium sulfate reduced the requirements for other muscle relaxants and sedatives and improved autonomic dysfunction (32). In a recent case series with two dogs, the time interval between acepromazine administration and muscle relaxation could be increased with magnesium supplementation (16). However, in animals, the dose range is empirical, and care should be taken to avoid adverse effects such as hypotension and arrhythmias.

In six dogs, signs of autonomic dysfunctions, such as hypertension, arrhythmias, bradycardia, or tachycardia, were present. Dysautonomia is a common complication of tetanus in human patients and is found in 11.8% of hospitalized patients in a cross-sectional study (33). In human patients with generalized tetanus, autonomic dysfunction is associated with poor prognosis (32). In the present study, cardiovascular autonomic dysfunction, such as premature ventricular contractions (*n* = 1), bradycardia (*n* = 1), or intermittent tachycardia (*n* = 2), was rarely observed. Autonomic dysfunction is rarely reported in animals with tetanus (11, 18, 34). However, autonomic dysfunction could be underrecognized due to inadequate monitoring and might be the unknown cause of death and was also suspected in the fatal cases in the present study. In the present study, the results from blood pressure measurements were available in 12 of 18 (66.7%) dogs with 41.7% of the dogs (5 of 12) showing severe hypertension. In contrast to this finding, primarily hypotension is described in previous case reports (9, 16). In human patients, clinical signs of autonomic dysfunction range from profound hypertension and tachycardia to bradycardia, arrhythmia, and hypotension (35). Elevated serum levels of norepinephrine, with spikes of epinephrine levels, cause a hyperdynamic circulatory state. These spikes increase up to 100 times normal, resembling levels occurring with pheochromocytoma in human patients (36). Excess acetylcholine may contribute to this autonomic dysfunction (37). Possible mechanisms of bradycardia are sudden withdrawal of catecholamines or increased vagal tone (35). In human patients, autonomic storms are often followed by severe resistant hypotension, bradycardia, and asystole and result in massive fluctuations in systemic vascular resistance index, with little change in cardiac index (37). In the present study, hypotension was not observed. Nevertheless, it is mandatory to continuously monitor electrocardiography for arrhythmia and control blood pressure regularly.

Respiratory complications occurred in 5 of 18 dogs (28%) with two dogs with aspiration pneumonia and three dogs with both aspiration pneumonia and laryngeal spasm. In a previous study, respiratory complications occurred at a similar rate and were observed in 26.4% of dogs with tetanus. In this study, poor outcome was associated with 14.2% of dogs that survived with respiratory complications compared with 94.8% of dogs without respiratory complications (10). Other studies also describe a poor outcome in cases with evidence of aspiration pneumonia (12, 14). In the present study, the management of dogs with aspiration pneumonia or laryngeal spasm required much more intensive care than the other dogs, and none of these dogs with respiratory compromise died.

Other rare complications in the present study were disseminated intravascular coagulation, urine retention, cystitis, and reduced gastrointestinal motility. Urinary tract infection is a known problem in cases with a long duration of catheterization, which might be necessary in dogs with urinary retention (38). Other rarely reported complications, such as coxo-femoral luxation (15) and hiatal hernia (11, 38, 39), were not observed in the present study. Reduced gastrointestinal motility, which was present in 3 of 18 (16.7%) dogs, has not been described in dogs with tetanus so far. In human patients, increased abdominal pressure and gastrointestinal stasis, even in patients who are awake, are known complications increasing the risk of aspiration (36).

The median hospitalization time of 8 days in the 16 surviving dogs is comparable to previous reports ranging from 0 to 30 days (12, 13). Median hospitalization time in surviving dogs was significantly associated with the grade of tetanus disease. Dogs with higher tetanus grade classification had a significantly longer median hospitalization time (18 days for grades III and IV).

The median time of improvement in clinical signs (11 days) was in a similar range compared with another retrospective analysis of 20 dogs (9.5 days and 5–12 days) (11). It has to be noted that the median time of improvement was slightly longer than the duration of hospitalization in the present study because dogs with milder and non-progressive signs of tetanus were discharged before clear improvement was observed. As TeNT binds irreversibly to axonal terminals and recovery is dependent on the synthesis of new presynaptic components and their transport to the distal axon, it is known that the duration of clinical improvement is approximately 2 weeks, and full recovery can require weeks to months (15, 40, 41). Muscle spasms can even be the result of permanent damage to inhibitory circuits within the CNS with unknown etiology (5). Long-term complications in humans after recovery, such as physical and psychological problems, are rarely reported (2, 42). In the present study, all surviving dogs recovered fully without any permanent deficits.

The binding of TeNT to neurons inhibits the release of inhibitory neurotransmitters, causing rigid muscle contractions (2). As TeNT can reach higher motor centers within the brain (the brainstem, midbrain, and hypothalamus) via retrograde intraneuronal transport, the subsequent prevention of GABA release can result in inappropriate activation of these motor centers and cause rapid eye movement (REM) sleep without characteristic muscle atonia, called rapid eye movement sleep behavior disorder (RBD) (13). In the present study, RBD was reported in all dogs in which information about sleep period was available. As electroencephalography (EEG) was not performed in all dogs, epileptic activity as a differential diagnosis to RBD cannot be excluded completely. As antiseizure drugs were used in 6/13 dogs and no improvement in episodes was reported, and indeed in one dog episodes deteriorated with a loading dose of levetiracetam and phenobarbital and episodes stopped when approaching and stimulating the dog, RBD was most likely. In one dog with severe episodes of RBD, an EEG was evaluated for confirmation. During a 3-h EEG, no epileptic paroxysmal discharges were observed, categorizing this disorder as non-epileptiform. RBD has been mentioned as “abnormal movements during sleep” in three dogs with tetanus (5). Sleep disturbances have also been reported by other authors (43, 44). A recent case series reported abnormal sleep behavior with violent epileptic-seizure-like RBD in 40% of dogs during recovery from tetanus (13). In a previous study, “abnormal movements during sleep” were already reported (5). In the present study, antiseizure drugs were ineffective in stopping these events, while RBD started during hospitalization; in previous reports, 75% of the RBDs started after discharge (13). The episodes of RBD stopped within weeks to months in all dogs in the present report.

Four dogs were immunized with tetanus toxoid subcutaneously. In human medicine, tetanus itself commonly does not induce antibody development (17). One older study on the Galápagos Islands describes the production of a naturally acquired antibody to tetanus toxin >0.01 IU/ml in humans and animals including four cows, two horses, two mules, and one dog (45). To the best of the authors' knowledge, no further reports evaluating antitoxoid antibodies, after disease or vaccination, are available in dogs. In the reported cases, 4 of 5 (80.0%) dogs did not develop an adequate amount of antitoxoid antibodies when tested within 36–149 days after the presentation at the clinic. It can be speculated that tetanus toxin alone is of low immunogenicity and that the neuronal receptor causes endocytosis of free toxin into the cells via the “A component” causing decreased contact of the toxin with the immune system. However, after 4 weeks of vaccination and booster vaccination, a rise in antibodies and adequate antibody concentration was present in all five dogs. As an adequate antibody titer, a serum antibody titer of > 0.01 IU/ml was considered, as this concentration is shown to be protective against tetanus in human medicine (46). After a second booster vaccination, a further rise in antibody titer was present in 3 of 5 dogs after 12 months, and the titer remained unchanged in the other two dogs. After 24 months, the antibody titer still rose in one dog and remained unchanged in the other dog (Table 2). In human medicine, the duration of immunity after vaccination was 11–14 years with antibodies being present for 64–74 years, measured using a double-antigen tetanus enzyme-linked immunosorbent assay (46). In dogs, the timing of further booster vaccinations has to be determined. The current guidelines of the World Small Animal Veterinary Association for the vaccination of dogs and cats currently do not list tetanus toxoid as a recommended vaccine but mention that tetanus is becoming more common, and “vaccine might be justified and commercially available” (47).

## 5. Limitations

The main limitation is the retrospective nature of the study. Therefore, not all data, such as on blood pressure and RBD, were available in all dogs, Furthermore, dogs were treated by different veterinarians. A standard operating procedure for tetanus patients was developed and adapted during the study period.

## 6. Conclusion

Tetanus is still a life-threatening disease but is also associated with a good prognosis if adequate management and monitoring can be ensured. In the present study with intensive, individual management and early institution of enteral nutrition, the mortality rate was in the lower range compared with previous publications. The benefit of vaccination is still questionable as tetanus is a rare disease in dogs, as this species is relatively resistant. After recovery from the disease, vaccination with a second booster dose 4 weeks later did result in an antibody increase. However, the usefulness of vaccination is still debatable.

## Data availability statement

The original contributions presented in the study are included in the article/Supplementary material, further inquiries can be directed to the corresponding author.

## Ethics statement

Ethical approval was not required for the studies involving animals in accordance with the local legislation and institutional requirements because the study involved medical records of the patients only. Written informed consent was not obtained from the owners for the participation of their animals in this study because the study involved medical records of the patients only.

## Author contributions

SD: conception and design of the study, data analysis, and manuscript preparation. CM and GW: data analysis, critical revision of the manuscript, and approved the submitted version. RS, AF, and KH: critical revision of the manuscript and approved the submitted version. RD: conception and design of the study, data analysis, statistical analysis, critical revision of the manuscript, and approved the submitted version. All authors contributed to the article and approved the submitted version.

## Footnotes

^1^Prism 5 for Windows (Graph Pad Software, Inc. San Diego, USA).

^2^ https://www.rki.de/DE/Content/Infekt/EpidBull/Merkblaetter/Ratgeber_Tetanus.html#doc2398266bodyText10

^3^ https://www.noahcompendium.co.uk/?id=-454714

^4^ https://www.openagrar.de/servlets/MCRFileNodeServlet/openagrar_derivate_00011385/Impfleitlinie_Wiederkaeuer_2018-03-01.pdf

## References

[1] Greene CE. Tetanus. In:Greene CE, editor. Infectious Diseases of the Dog and Cat (4th ed. Amsterdam: Elsevier Saunders. (2012). p. 423–31.

[2] Cook TM Protheroe RT Handel JM. Tetanus: a review of the literature. Br J Anaesth. (2001) 87:477–87. 10.1093/bja/87.3.477 11517134

[3] Popoff MR. Tetanus in animals. J Vet Diagn Invest. (2020) 32:1–8. 10.1177/1040638720906814 PMC7081504 32070229

[4] Humeau Y Doussau F Grant NJ Poulain B. How botulinum and tetanus neurotoxins block neurotransmitter release. Biochemie. (2000) 82:427–46. 10.1016/S0300-9084(00)00216-9 10865130

[5] Burkitt JM Sturges BK Jandrey KE Kass PH. Risk factors associated with outcome in dogs with tetanus: 38 cases (1987–2005). J Am Vet Med Assoc. (2007) 230:76–83. 10.2460/javma.230.1.76 17199496

[6] Rossetto O Montecucco C. Tables of toxicity of botulinum and tetanus neurotoxins. Toxins. (2019) 11:686. 10.3390/toxins11120686 31771110 PMC6950492

[7] Galazka AM,. The Immunological Basis for Immunization, Module 3. Tetanus: WHO. (2001). Available online at: https://apps.who.int/iris/bitstream/handle/10665/58891/WHO-EPI-GEN-93.13-mod3-eng.pdf?sequence=51&isAllowed=y

[8] Coleman ES. Clostridial neurotoxins: tetanus and botulism. Comp Cont Educ Small Anim Pract. (1998) 20:1089.

[9] Fawcett A Irwin P. Diagnosis and treatment of generalized tetanus in dogs. In Pract. (2014) 36:482–93. 10.1136/inp.g6312

[10] Guedra M Humm K Cortellini S. Respiratory complications in dogs with tetanus: a retrospective study of 53 cases. Can Vet J. (2021) 62:1202–6. 34728847 PMC8543695

[11] Acke E Jones BR Breathnach R McAllister H Mooney CT. Tetanus in the dog: review and a case-report of concurrent tetanus with hiatal hernia. Ir Vet J. (2004) 57:593–97. 10.1186/2046-0481-57-10-593 21851651 PMC3113811

[12] Bandt C Rozanski E Steinberg T Shaw S. Retrospective study of tetanus in 20 dogs: 1988–2004. J Am Anim Hosp Assoc. (2007) 43:143–148. 10.5326/0430143 17473020

[13] Shea A Hatch A Risico L Beltran E. Association between clinically probable REM sleep behavior disorder and tetanus in dogs. J Vet Intern Med. (2018) 32:2029–36. 10.1111/jvim.15320 30315605 PMC6272037

[14] Zitzl J DyckersJ Güssow A Hazuchova K Lehmann H. Survival in canine tetanus: retrospective analysis of 42 cases (2006–2020). Front Vet Sci. (2022) 15:1015569. 10.3389/fvets.2022.1015569 36590798 PMC9797805

[15] Adamantos S Boag A. Thirteen cases of tetanus in dogs. Vet Rec. (2007) 161:298–302. 10.1136/vr.161.9.298 17766808

[16] Papageorgiou V Kazakos G Anagnostou T Polizopoulou Z. The role of magnesium in the management of acute and long-term symptoms caused by tetanus in two dogs. Topics Comp Animal Med. (2021) 44:100535. 10.1016/j.tcam.2021.100535 33933700

[17] Peter R Nunley MK Demetriades D Velmahos G Doucet JJ. Tetanus and trauma: a review and recommendations. J Trauma. (2005) 58:1082–8. 10.1097/01.TA.0000162148.03280.02 15920431

[18] Linnenbrink T McMichael M. Tetanus: pathophysiology, clinical signs, diagnosis, and update on new treatment modalities. J Vet Emerg Crit Care. (2006) 16:199–207. 10.1111/j.1476-4431.2006.00192.x

[19] Medicine Healthcare Products Regulatory Agency. Non WHO Material, Tetanus Antitoxin, Equine, For Bioassay, 3rd British Standard. (2012) Available online at: https://www.nibsc.org/documents/ifu/60-013.pdf

[20] Mayousse V Soete C Jeandel A. Suspected generalized neonatal tetanus in a litter of puppies. J Am Anim Hosp Assoc. (2023) 59:51–5. 10.5326/JAAHA-MS-7246 36584314

[21] Firor WM. Intrathecal administration of tetanus antitoxin. Arch Surg. (1940) 41:299–307. 10.1001/archsurg.1940.01210020095011

[22] Soubasis N Koutinas AF Saridomichelakis MN Polizopoulou ZS. Tetanus in the dog: a study of 6 cases. Eur J Comp Animal Pract. (2002) 12:19–23.

[23] Steinman A Haik R Elad D Sutton GA. Intrathecal administration of tetanus antitoxin to three cases of tetanus in horses. Equine Vet Educ. (2000) 12:237–40. 10.1111/j.2042-3292.2000.tb00049.x

[24] Sedaghatian MR. Intrathecal serotherapy in neonatal tetanus - controlled trial. Arch Dis Child. (1979) 54:623–5. 10.1136/adc.54.8.623 389175 PMC1545797

[25] Vakil BJ Armitage P Clifford RE Laurence DR. Therapeutic trial of intracisternal human tetanus immunoglobulin in clinical tetanus. T Roy Soc Trop Med H. (1979) 73:579–83. 10.1016/0035-9203(79)90058-0 583378

[26] Bhandari B Ajmera NK Jagetiya PR. Intrathecal anti-tetanus serum in management of tetanus neonatorum. Indian J Med Res. (1980) 72:685–7. 6894134

[27] Abrutyn E Berlin JA. Intrathecal therapy in tetanus. A meta-analysis. J Am Med Assoc. (1991) 226:2262–7. 10.1001/jama.1991.03470160094039 1833565

[28] Farrar JJ Yen LM Cook T Fairweather N Binh N Parry J. Tetanus. J Neurol Neurosurg Psychiat. (2000) 69:292–301. 10.1136/jnnp.69.3.292 10945801 PMC1737078

[29] Bruce RB Turnbull LB Newman JH. Metabolism of methocarbamol in rat, dog and human. J Pharm Sci. (1971) 60:104–6. 10.1002/jps.2600600120 5548215

[30] Sahal M Haydardedeoglu AE Cingi CC. Generalized tetanus in a dog after ovariohysterectomy. Kafkas Univ Vet Fak Derg. (2011) 17:877.

[31] Taruo A Beltran E Cherubini GB Coelho AT Wessmann A Driver CJ. Metronidazole-induced neurotoxicity in 26 dogs. Aust Vet J. (2018) 96:495–501. 10.1111/avj.12772 30478843

[32] Thwaites CL Yen LM Loan HT Thuy TTD Thwaites GE Stepniewska K. Magnesium sulphate for treatment of severe tetanus: a randomize controlled trial. Lancet. (2006) 368:1436–43. 10.1016/S0140-6736(06)69444-0 17055945

[33] Derbie A Amdu A Alamneh A. Clinical profile of tetanus patients attended at Felege Hiwot Referral Hospital, Northwest Ethiopia: a retrospective cross-sectional study. Springerplus. (2016) 5:892. 10.1186/s40064-016-2592-8 27386340 PMC4923016

[34] Panciera DL Baldwin CJ Keene BW. Electrocardiographic abnormalities associated with tetanus in two dogs. J Am Vet Med Assoc. (1988) 192:225–7. 3350751

[35] Sharma N Li S Sravanthi MV Kazmierski D Wang Y Sharma A. Tetanus complicated by dysautonomia: a case report and review of management. Case Rep Crit Care. (2021) 88:42522. 33815849 10.1155/2021/8842522 PMC7987407

[36] Freshwater-Turner D Udy A Lipman J. Autonomic dysfunction in tetanus - what lessons can be learned with specific reference to alpha-2 agonists? Anaesthesia. (2007) 62:1066–70. 10.1111/j.1365-2044.2007.05217.x 17845661

[37] Dolar D. The use of continuous atropine infusion in the management of severe tetanus. Intens Care Med. (1992) 18:26–31. 10.1007/BF01706422 1578043

[38] Ham L Van Bree H. Conservative treatment of tetanus associated with hiatal hernia and gastro-oesophageal reflux. J Small Anim Pract. (1992) 33:289–94. 10.1111/j.1748-5827.1992.tb01146.x

[39] Dieringer TM Wolf AM. Esophageal hiatal hernia and megaesophagus complicating tetanus in two dogs. J Am Vet Med Assoc. (1991) 199:87–9. 1885336

[40] Bleck TP Brauner JS. Tetanus. In:Eds Scheld WM Whitley RJ Durack DT, editor. Infections of the Central Nervous System, 2nd ed. Philadelphia: Lippincourt-Raven. (1997). p. 629–653.

[41] Verma A Solbrig MV. Infections of the nervous system. In:Bradley WG Daroff RB Fenichel GM Jankovic J, editors. Neurology in Clinical Practice: The Neurological Disorders, 4th ed. Philadelphia: Butterworth Heinemann. (2004) p. 1510.

[42] Illis LS Taylor FM. Neurological and electroencephalographic sequelae of tetanus. Lancet. (1971) 7704:826–30. 10.1016/S0140-6736(71)91496-6 4102526

[43] Hanson CJ. Tetanus in a dog: a case report. Vet Rec. (1982) 110:336–7. 10.1136/vr.110.14.336 7080424

[44] Zontine WJ Uno T. Tetanus in a dog. (A case report). Vet Med Small Anim Clin. (1968) 63:341–4. 5184659

[45] Veronesi R Bizzini B Focaccia R. Naturally acquired antibodies to tetanus toxin in humans and animals from the galapagos islands. J Infect Dis. (1983) 147:308–11. 10.1093/infdis/147.2.308 6827147

[46] Hammarlund E Thomas A Poore EA Amanna IJ Rynko AE Mori M. Durability of vaccine-induced immunity against tetanus and diphtheria toxins: a cross-sectional analysis. Clin Infect Dis. (2016) 62:1111–8. 10.1093/cid/ciw066 27060790 PMC4826453

[47] Day JM Horzinek MC Schultz RD Squires RA WSAVA. Guidelines for vaccination of dogs and cats. JSAP. (2016) 57:E1–E45. 10.1111/jsap.2_12431 PMC7166872 26780857

