# Supplementary material for: Retrospective study of tetanus in 18 dogs—Causes, management, complications, and immunological status
Source: Front Vet Sci. 2023 Nov 2;10:1249833. doi: 10.3389/fvets.2023.1249833 (PMC10651760; doi:10.3389/fvets.2023.1249833)
Supplement: Supplementary file 1 [file Data_Sheet_1.pdf]

Supplementary file 1: Information about breed, body weight, occurrence of clinical signs, tetanus grade at presentation, progression and improvement of clinical signs, hospitalization time, and outcome in 18 dogs with tetanus.

Case No	Breed (sex)	Body weight (kg)	Age (mo)	Time (days) of occurrence of clinical signs after wound was detected	Time (days) of wound detection till presentation	Tetanus grade at presentation	Progression to maximum tetanus grade	Improvement of clinical signs after hospitalization (days)	Hospitalization (days)	Outcome
**1**	Labrador retriever (MN)	28.5	28	4	9	II	Grade IV after surgery (2 days)	10	23	Survived
**2**	Mixed-breed (FI)	20.0	61	14	16	I	Stable	Not documented	0∬	Survived
**3**	Dachshund-mix (FN)	10.0	83	8	13	III	Grade IV within 3 days	Died	3	Died
**4**	Great Dane (FI)	50.0	12	7	11	III	Grade IV after surgery (1 day)	14	24	Survived
**5**	Rhodesian Ridgeback (MI)	30.0	48	%	%	III	Stable	Died	3	Died
**6**	Elo (MI)	24.0	60	5	17	II	%	%	2	Survived

**7**	Labrador-mix (FI)	29.0	26	27	28	II	Grade IV within 4 days	12	19	Survived
**8**	Labrador (FN)	26.0	79	14	20	II + hypothermia	Stiffness mildly worse after anesthesia (1 day)	7	9	Survived
**9**	Boxer (MI)	19.0	10	%	%	II	Stable	5	7	Survived
**10**	Mixed-breed (FN)	26.0	59	1	4	II	Grade IV after surgery, laryngeal spasm (1 day)	11	18	Survived
**11**	Short-haired Collie (MI)	21.5	18	5	16	I	Stable	Was stable	4	Survived
**12**	American Pocket Bully (FI)	29.0	45	4	6	II	Grade IV after surgery (1 day)	13	14	Survived
**13**	Boxer-mix (FN)	33.0	17	6	10	II	Stable	12$^{\triangledown}$	3	Survived

**14**	Dutch Shepherd dog (MI)	25.0	21	%	%	II	Stable	15▽	4	Survived
**15**	Jack Russel Terrier (MI)	5.0	5	%	%	III (spasms)	Stable	13▽	6	Survived
**16**	Border Collie (MN)	23.5	90	%	%	II	Minimal progressive, but stable grade II	4	5	Survived
**17**	American Staffordshire Terrier (MN)	25.0	38	3	4	II	Grade III within 4 days	11	26	Survived
**18**	Mixed-breed (FN)	13.0	31	7	12	II	Grade III within 5 days	10	17	Survived

%: unknown; MN: male neutered; ME: male intact; FE: female intact; FN: female neutered, mo: month, ∬management only at home, ▽clear improvement of signs not documented, time of voluntary food intake applied.

Supplementary file 2: Site of infection, radiographic evaluation of the infected site, isolated bacteria and surgical procedure performed in eighteen dogs with tetanus disease.

Case No	Site of infection	Bacteria isolated	Radiographic evaluation	Surgical procedure
1	Ph II left TL	*Staphylococcus simulans*, *Enterococcus casseliflavus*, no growth of obligate anaerobic bacteria (surgery quality control swab)	Radiographic evidence for osteomyelitis of the distal phalanx with soft tissue swelling	Amputation of the digit at the distal level of the metacarpal bone via osteotomy
2	Right TL*	Not performed	Without diagnostic findings	Not performed
3	Ph IV left PL	None at surgery (quality control swab)	Radiographic evidence for osteomyelitis of the distal phalanx with soft tissue swelling	Amputation of the digit at the distal level of the metacarpal bone via osteotomy
4	Ph II right PL	Not performed	Radiographic evidence for osteomyelitis of the distal phalanx, soft tissue swelling, displacement of claw	Amputation of the digit at the distal level of the metacarpal bone via osteotomy
5	%	Not performed	Not performed	Not performed
6	Ph IV right TL	None at surgery (quality control swab)	Radiographic evidence for osteomyelitis and pathologic fracture of the distal phalanx, gas inclusion in the claw, soft tissue swelling	Amputation of the digit at the distal level of the metacarpal bone via osteotomy
7	Ph III left TL	Not performed	Without diagnostic findings	Not performed

8	%	Not performed	Not performed	Not performed
9	Ph III/IV right TL	Not performed	Without diagnostic findings	Not performed
10	Ph II right TL	*Citrobacter freundii*, *Enterococcus faecium*; anaerobic culture: *Clostridium sporogenes*	Radiographic evidence for gas inclusion of the distal phalanx, soft tissue swelling	Amputation of the digit at the distal level of the metacarpal bone via osteotomy
11	Ph III right PL, Ph III/ IV left PL	Out of four swabs from necrotic tissue: *Escherichia coli; Citrobacter* species*; Pasteurella canis; Streptococcus canis; Staphylococcus pseudintermedius; Staphylococcus aureus; Clostridium tertium*; further Bacteria (differentiation without result); anaerobic cultures: *Clostridium sporogenes, Paeniclostridium ghonii; Clostridium* species; *Peptostreptococcus canis. Clostridium tetani* not excluded due to overgrown plates.	Abrasion of all claws, fracture of distal phalanx III right PL/ II left PL, radiographic evidence for osteomyelitis, soft tissue swelling	Amputation of the distal phalanx III right PL/ IV left PL, amputation of the digit III left PL at the level of the metacarpophalangeal joint
12	Ph III right TL	***Clostridium tetani*** and other *Clostridia* out of necrotic tissue, *Staphylococcus pseudintermedius* und *Citrobacter freundii* out of control swab at surgery.	Soft tissue swelling	Amputation of the digit at the distal level of the metacarpal bone via osteotomy
13	Ph II left TL	*Enterobacter* species out of control swab at surgery.	Soft tissue swelling, fracture of the tip of the distal phalanx	Amputation of the digit at the distal level of the metacarpal bone via osteotomy
14	Ph V right PL, Ph II left PL	Not performed	Not performed	Not performed
15	teething	Not performed	Not performed	Not performed

16	wound proximal PL*	%	Not done	Not done
17	PhV left TL	*Proteus* spp; anaerobic culture: none	Radiographic evidence for osteomyelitis and gas inclusion of the distal phalanx, soft tissue swelling	Amputation of the digit at the distal level of the metacarpal bone via osteotomy
18	Ph I left PL	%	Not performed	Not performed

%: unknown; *not further identified; Ph: phalanx; PL: pelvic limb, TL: thoracic limb.

Supplementary file 3: Different treatment protocols of each patient with adverse effects and complications listed, as wells as time to spontaneous food uptake and removal of the percutaneous endoscopic gastropexy tube and information about rapid eye movement behavior disorder.

Case No.	Treatment	Adverse effects of treatment	Nutrition	Time to spontaneous food ingestion (days after presentation)/PEG tube removed (days after presentation)	Complications	RBD
1	• Antitoxin intravenous (350 IU/kg)<br>• CVC placement<br>• Sedation: acepromazine CRI, midazolam CRI, dexmedetomidine CRI, propofol boli<br>• Antiseizure drug: phenobarbital<br>• Analgesia: buprenorphine<br>• Antibiotics: metronidazole, amoxicillin/clavulanic acid	Dysphoria after midazolam	PEG-tube	%/22	BP %<br>PVC´s<br>Aspiration pneumonia	Severe

	• Antiemetics: maropitant, metoclopramide<br>• Muscle relaxant: methocarbamol<br>• Omeprazole					
**2**	• Antitoxin intramuscular (250 IU/kg)<br>• Sedation: acepromazine boli<br>• Antibiotics: metronidazole<br>• Muscle relaxant: methocarbamol	%	oral	%	BP %	%
**3**	• Sedation: dexmedetomidine CRI, acepromazine CRI, propofol boli<br>• Antiseizure drug: phenobarbital<br>• Analgesia: buprenorphine, metamizole<br>• Antibiotics: metronidazole<br>• Muscle relaxant: methocarbamol<br>• Omeprazole	%	PEG-tube	NN	BP %<br>Death of unknown cause	%
**4**	• CVC placement<br>• Sedation: midazolam CRI, dexmedetomidine boli and CRI<br>• Analgesia: methadone CRI, later buprenorphine	%	PEG-tube	%/32	BP 127/58 (82) mmHg<br>Aspiration pneumonia<br>DIC	%

	• Antibiotics: metronidazole, amoxicillin/clavulanic acid, marbofloxacin<br>• Antiemetics: maropitant, metoclopramide<br>• Muscle relaxant: methocarbamol<br>• Omeprazole<br>• Magnesium sulfate				Severe inflammation of the toe after surgery	
**5**	• Sedation: dexmedetomidine CRI and boli, propofol boli<br>• Analgesia: buprenorphine<br>• Antibiotics: metronidazole<br>• Antiemetic: maropitant<br>• Muscle relaxant: methocarbamol<br>• Omeprazole	%	oral	%	BP %<br>Home management after 3 days, death of unknown cause at home	%
**6**	• Antitoxin intravenous (375 IU/kg)<br>• Sedation: dexmedetomidine CRI and boli<br>• Analgesia: fentanyl CRI, metamizole<br>• Antibiotics: metronidazole<br>• Antiemetics: maropitant	%	PEG-tube	%	BP %	%

	• Muscle relaxant: methocarbamol<br>• Omeprazole					
**7**	• PICC, for parenteral nutrition CVC placement<br>• Sedation: acepromazine CRI, dexmedetomidine CRI, propofol CRI, butorphanol CRI, Midazolam bolus<br>• Antiseizure drug: phenobarbital, levetiracetam<br>• Analgesia: fentanyl CRI, methadone<br>• Antibiotics: metronidazole, amoxicillin/clavulanic acid, marbofloxacin<br>• Antiemetics: maropitant, metoclopramide boli<br>• Muscle relaxant: methocarbamol<br>• Omeprazole<br>• Ranitidine<br>• Chlorphenamine<br>• O_2 supplementation	• Dysphoria after midazolam<br>• Vomiting despite metoclopramide CRI<br>• Escape beats and bradycardia during buprenorphine	PEG-tube	23/26	• BP 220/108 (146) mmHg, treated with amlodipine<br>• Laryngeal spasm, intubation and propofol CRI<br>• Aspiration pneumonia<br>• Bradycardia (25 – 35 bpm) and escape beats<br>• Hypalbuminemia (18.2 g/l) despite PEG-tube feeding, stiffness deteriorated<br>• Reduced gastrointestinal motility<br>• Vomitus despite metoclopramide CRI, stopped with metoclopramide boli subcutaneously	Severe, verified with EEG

					• $etCO_2$ > 60 mmHg and SpO_2 < 90%without oxygen supplementation<br>• Urine retention, cystitis	
**8**	• CVC placement<br>• Sedation: acepromazine CRI, dexmedetomidine CRI, propofol boli<br>• Analgesia: methadone<br>• Antibiotics: metronidazole, ampicillin<br>• Antiemetics: maropitant, metoclopramide CRI<br>• Muscle relaxant: methocarbamol<br>• Omeprazole		PEG-tube	9/14	• BP: WNL	Mild
**9**	• Antitoxin intravenous (300 IU/kg)<br>• CVC placement<br>• Sedation: acepromazine CRI<br>• Analgesics: methadone<br>• Antibiotics: metronidazole		PEG-tube	12/19	BP 202/98 (133) mmHg, treated with amlodipine	Mild

	• Antiemetics: metoclopramide CRI<br>• Muscle relaxant: methocarbamol					
**10**	• Antitoxin intravenous (300 IU/kg)<br>• CVC placement<br>• Sedation: acepromazine CRI, propofol CRI<br>• Antiseizure drug: levetiracetam<br>• Analgesia: methadone<br>• Antibiotics: metronidazole, amoxicillin/clavulanic acid<br>• Antiemetics: metoclopramide CRI<br>• Muscle relaxant: methocarbamol<br>• Ranitidine	• SpO_2 < 90% during propofol CRI (oxygen supplementation)<br>• Respiratory arrest with low dose dexmedetomidine	PEG-tube	27/34	• BP 192/111 (155) mmHg, treated with amlodipine<br>• Aspiration pneumonia<br>• Laryngeal spasm prior to surgery, after manipulation<br>• Reduced gastrointestinal motility<br>• Lateralized stiffness (left side more than right side)	Severe
**11**	• Antitoxin intravenous (200 IU/kg)<br>• Analgesia: grapiprant<br>• Antibiotics: metronidazole, amoxicillin/clavulanic acid<br>• Antiemetics: maropitant<br>• Muscle relaxant: methocarbamol	none	oral	NN	BP %<br>none	Mild

**12**	• Antitoxin subcutaneously (690 IU/kg, in another clinic)<br>• CVC placement<br>• Sedation: acepromazine CRI, propofol CRI, dexmedetomidine CRI<br>• Antiseizure drug: levetiracetam, gabapentin<br>• Analgesia: methadone, metamizole<br>• Antibiotics: metronidazole, amoxicillin/clavulanic acid, marbofloxacin<br>• Antiemetics: maropitant, metoclopramide<br>• Muscle relaxant: methocarbamol<br>• Chlorphenamine	• Vomitus during dexmedetomidine CRI<br>• SpO_2 < 80% during propofol CRI	PEG-tube	30/34	Laryngeal spasm and tongue and laryngeal swelling after anesthesia/surgery<br>Aspiration pneumonia<br>SpO_2 < 80% without oxygen supplementation, increased to 90% with oxygen<br>BP elevated 189/100 (149) mmHg, treated with amlodipine<br>Intermittent tachycardia<br>Reduced gastrointestinal motility<br>Fever, treated with Marbofloxacin	Severe
**13**	• Antitoxin intravenous (200 IU/kg)<br>• Sedation: acepromazine boli<br>• Analgesia: methadone, buprenorphine at day 2, metamizole<br>• Antibiotics: metronidazole, ampicillin	none	PEG-tube	12/18	BP WNL<br>none	Mild

	• Antiemetics: maropitant, metoclopramide<br>• Muscle relaxant: Methocarbamol					
**14**	• Antitoxin intravenous (300 IU/kg)<br>• CVC placement<br>• Sedation: acepromazine boli<br>• Antiseizure drug: levetiracetam<br>• Analgesia: methadone<br>• Antibiotics: metronidazole<br>• Antiemetics: maropitant<br>• Muscle relaxant: methocarbamol	• Tremor after butorphanol, stopped with midazolam	PEG-tube	15/21	none<br>BP 130/80 (95) mmHg	Mild
**15**	• Antitoxin intravenous (250 IU/kg)<br>• Sedation: acepromazine boli<br>• Analgesia: methadone for 24 h, Metamizole<br>• Antibiotics: metronidazole, amoxicillin/clavulanic acid<br>• Muscle relaxant: methocarbamol	none	PEG-tube	13/18	BP 120/60 (82) mmHg<br>intermittent tachycardia	Mild
**16**	• Antitoxin intravenous (300 IU/kg)<br>• Sedation: acepromazine boli	none	PEG	15/21	BP 148/65 (98)	Severe

	• Analgesia: methadone<br>• Antibiotics: metronidazole<br>• Muscle relaxant: methocarbamol				172/68 (104)	
**17**	• Antitoxin intravenous (200 IU/kg)<br>• CVC placement<br>• Sedation: acepromazine CRI, dexmedetomidine CRI, propofol CRI<br>• Analgesia: methadone, buprenorphine<br>• Antiseizure drugs: levetiracetam, gabapentin<br>• Antibiotics: metronidazole, amoxicillin/clavulanic acid<br>• Antiemetic: maropitant<br>• Muscle relaxant: methocarbamol<br>• Magnesium sulfate<br>• Oxygen supplementation	Bradycardia	PEG	19/26	BP 156/83 (118)<br>urinary retention	mild
**18**	• Antitoxin intravenous (200 IU/kg)<br>• CVC placement<br>• Sedation: acepromazine CRI	none	Low Profile PEG	19/25	BP 145/88 (112)	severe

	• Analgesia: methadone, buprenorphine<br>• Antiseizure drugs: gabapentin<br>• Antibiotics: metronidazole, amoxicillin/clavulanic acid<br>• Antiemetic: maropitant<br>• Muscle relaxant: methocarbamol					

BP: blood pressure; % not investigated/unknown; CRI: constant rate infusion; CVC: central venous catheter; PVC: premature ventricular contractions; DIC: disseminated intravascular coagulation; bpm: beat per minute; PEG-tube: percutaneous endoscopically placed gastrotomy tube, PICC: peripherally inserted central venous catheter; RBD: rapid eye movement sleep behavior disorder; EEG: electroencephalography; WNL: within normal limits; NN: not applicable.
